# Characterization of transferrin receptor-mediated endocytosis and cellular iron delivery of recombinant human serum transferrin from rice (*Oryza sativa* L.)

**DOI:** 10.1186/1472-6750-12-92

**Published:** 2012-11-30

**Authors:** Deshui Zhang, Hsin-Fang Lee, Steven C Pettit, Jennica L Zaro, Ning Huang, Wei-Chiang Shen

**Affiliations:** 1 Ventria Bioscience, 320 East Vine Drive, Fort Collins, CO, 80524, USA; 2 University of Southern California, School of Pharmacy, 1985 Zonal Avenue, Los Angeles, CA, 90089-9121, USA; 3 InVitria, 320 East Vine Drive, Fort Collins, CO, 84524, USA

**Keywords:** Recombinant human serum transferrin, Transferrin receptor, Endocytosis, Cell growth and proliferation, Antibody production

## Abstract

**Background:**

Transferrin (TF) plays a critical physiological role in cellular iron delivery via the transferrin receptor (TFR)-mediated endocytosis pathway in nearly all eukaryotic organisms. Human serum TF (hTF) is extensively used as an iron-delivery vehicle in various mammalian cell cultures for production of therapeutic proteins, and is also being explored for use as a drug carrier to treat a number of diseases by employing its unique TFR-mediated endocytosis pathway. With the increasing concerns over the risk of transmission of infectious pathogenic agents of human plasma-derived TF, recombinant hTF is preferred to use for these applications. Here, we carry out comparative studies of the TFR binding, TFR-mediated endocytosis and cellular iron delivery of recombinant hTF from rice (rhTF), and evaluate its suitability for biopharmaceutical applications.

**Result:**

Through a TFR competition binding affinity assay with HeLa human cervic carcinoma cells (CCL-2) and Caco-2 human colon carcinoma cells (HTB-37), we show that rhTF competes similarly as hTF to bind TFR, and both the TFR binding capacity and dissociation constant of rhTF are comparable to that of hTF. The endocytosis assay confirms that rhTF behaves similarly as hTF in the slow accumulation in enterocyte-like Caco-2 cells and the rapid recycling pathway in HeLa cells. The pulse-chase assay of rhTF in Caco-2 and HeLa cells further illustrates that rice-derived rhTF possesses the similar endocytosis and intracellular processing compared to hTF. The cell culture assays show that rhTF is functionally similar to hTF in the delivery of iron to two diverse mammalian cell lines, HL-60 human promyelocytic leukemia cells (CCL-240) and murine hybridoma cells derived from a Sp2/0-Ag14 myeloma fusion partner (HB-72), for supporting their proliferation, differentiation, and physiological function of antibody production.

**Conclusion:**

The functional similarity between rice derived rhTF and native hTF in their cellular iron delivery, TFR binding, and TFR-mediated endocytosis and intracellular processing support that rice-derived rhTF can be used as a safe and animal-free alternative to serum hTF for bioprocessing and biopharmaceutical applications.

## Background

Iron is an essential element for cell growth and metabolism. The major vehicle for iron delivery is serum transferrin (TF), which plays a crucial role in tightly regulating cellular iron uptake, transport, and utilization in nearly all eukaryotes. The mechanism by which TF overcomes the dual challenges of iron deficiency and overload in cells is via a TF/TF receptor (TFR)-mediated endocytotic process [1]. When TF is free of iron (apo-TF), it can bind two iron molecules (diferric TF or holo-TF) at the extracellular pH of 7.4. The resultant holo-TF binds to TFR with a greater affinity than apo-TF, where two diferric TF molecules will bind to the homodimeric TFR on the cell surface [2]. This TF–TFR complex is then endocytosed into the early endosome, where the acidic environment (pH 5.5) triggers the conformational change of TF–TFR complex and the subsequent release of iron from TF. Finally, the TF–TFR complex is recycled to the cell surface, where the lower affinity of apo-TF for TFR at the neutral extracellular pH will dissociate the complex and release the TF for re-use [3].

TF has been successfully used or being evaluated in a wide range of important biopharmaceutical applications. TF has been widely used as an important supplement in culture medium for various mammalian cells and stem cells because of the absolute requirement of iron for cellular growth and proliferation [4,5]. TF has also been actively pursued as a drug delivery vehicle due to its unique receptor-mediated endocytosis pathway as well as its added advantages of being biodegradable, nontoxic, and nonimmunogenic [6-9]. Moreover, TF is also exploited for oral delivery of protein-based therapeutics [10,11], as TFR is abundantly expressed in human gastrointestinal (GI) epithelium and TF is resistant to proteolytic degradation [10,12].

With the increasing concerns over the risk of transmission of infectious pathogenic agents from the use of human and animal plasma-derived TFs in both cell culture and drug delivery applications [13-15], rhTF has long been pursued in a variety of expression systems [16-24]. However, expression of fully functional TF has been proven to be challenging largely due to hTF’s complicated structural characteristics including 19 disulfide bonds and two homologous lobes (N-lobe and C-lobe). We have achieved a high level of expression yield of rhTF in rice (*Oryza sativa* L.). Expression yield is 40% of total soluble protein or 1% seed dry weight (10 g/kg) [25]. The rice-derived rhTF is shown to be biochemically and structurally similar to hTF and mammalian cell-derived rhTF, and able to bind Fe^3+^ tightly yet reversibly [15]. In the present work, we have characterized rice-derived rhTF’s TFR-binding, TFR-mediated endocytosis and intracellular processing, and its cellular iron delivery to diverse mammalian cells through various cell-based assays.

## Results

### Comparison of TFR-binding affinity of rhTF and hTF

**Figure 1:**
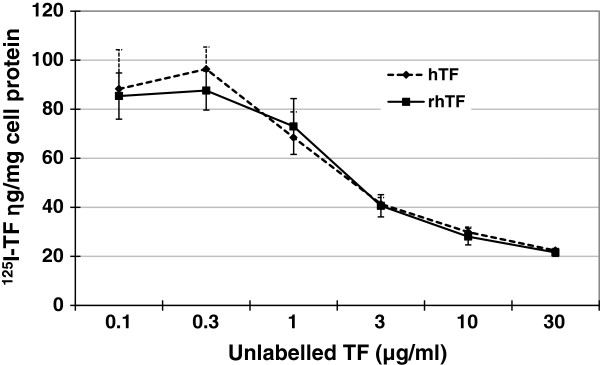
**TFR**-**competition binding assay of rhTF and hTF in Caco**-**2 cells.** Different concentrations of rhTF or hTF mixed with 1 μg/ml of ^125^I-hTF were added to confluent Caco-2 cells and incubated at 4°C for 1 hr. Cells were then washed with cold PBS, solubilized with 1 N NaOH, and the amount of radiolabelled hTF in cell lysate was determined. The total cellular protein in the cell lysate was measured, and used to normalize the data to ng of hTF per mg cell protein (“ng/mg cell protein”). Data is represented as average with error bars indicating standard deviation, n = 3.

We compared the binding affinity of rhTF and hTF to the TFR by a competition binding affinity assay. A series of increasing concentrations of rhTF or hTF were mixed with a fixed concentration (1 μg/ml) of ^125^I-labeled hTF, and added to confluent Caco-2 cells for competition binding to TFR (Figure 1). The binding of ^125^I-hTF in Caco-2 cells is apparently inhibited with the addition of one-fold excess or more of unlabeled rhTF or hTF, indicating that both rhTF and hTF compete with ^125^I-hTF to bind to TFR. Moreover, rhTF and hTF show the same extent of dose-dependent inhibition of the binding of ^125^I-hTF to TFR in Caco-2 cells. This result indicates that rhTF competes similarly as hTF to the TFR binding.

**Figure 2:**
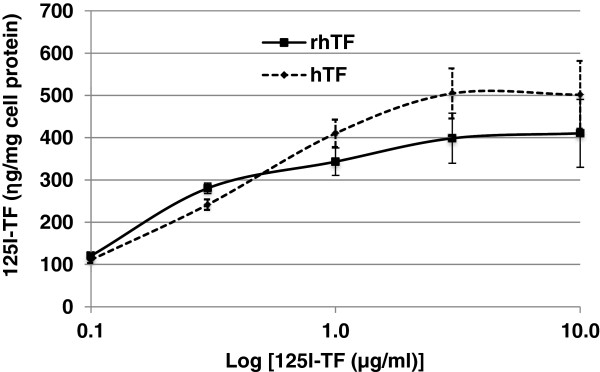
**TFR-binding affinity of rhTF and hTF in HeLa cells.** Different concentrations of ^125^I-labeled hTF or rhTF were added to confluent HeLa cells and incubated at 4°C for 2 hr. Cells were then washed with cold PBS, solubilized with 1 N NaOH, and the amount of radiolabelled hTF or rhTF in cell lysate was determined. The total cellular protein in the cell lysate was measured, and used to normalize the data to ng of hTF per mg cell protein (“ng/mg cell protein”). Data is represented as average with error bars indicating standard deviation, n = 3.

To directly compare rhTF’s TFR binding affinity with that of hTF, the TFR binding capacity (B_max_) and equilibrium dissociation constant (K_d_) values of both rhTF and hTF were determined using saturation radioligand binding assays in HeLa cells (Figure 2, Table 1). Both rhTF and hTF’s TFR binding in HeLa cells exhibits a similar dose-dependent TFR binding profile (Figure 2). While the K_d_ values of rhTF and hTF are significantly different (Table 1), they are within the normal range for hTF (K_d_ = 2 – 8 nM) [26-28]. The difference of B_max_ values of hTF and rhTF appears small but statistically significant (Table 1). It is unknown if these B_max_ values also fall into a normal range because the B_max_ value is cell line dependent and there is no published B_max_ values for hTF in HeLa cells. Nevertheless, these results demonstrate that both the TFR binding capacity and dissociation constant of rhTF are comparable to that of hTF.

**Table 1:** TFR binding parameters of TF proteins in HeLa cells

**TFR binding parameters**	**hTF**	**rhTF**	***P-*Value**
B_max_ (pmol/mg cell protein)^1^	6.78 ± 0.21	5.62 ± 0.20	0.0023^b^
K_d_ (nM)^2^	4.42 ± 0.58	2.61 ± 0.46	0.0133^a^

^1^B_max_ = maximum number of binding sites.

^2^K_d_ = equilibrium dissociate constant.

^a^*P*-value < 0.05.

^b^*P*-value < 0.01.

### TFR-mediated endocytosis of rhTF

#### Comparison of rhTF endocytosis in Caco-2 and HeLa cells

**Figure 3:**
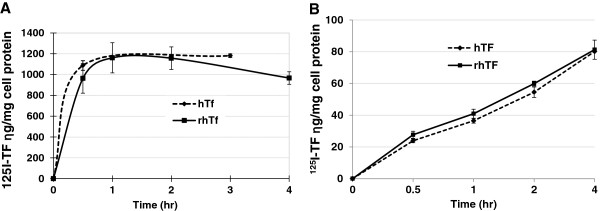
**Comparison of uptake kinetics of rhTF and hTF in HeLa and Caco**-**2 cells. A**. Uptake kinetics of rhTF and hTF in HeLa cells. One μg/ml of ^125^I-labeled rhTF was added to confluent HeLa cells, and incubated at 37°C for 0.5, 1, 2, and 4 hr. In a comparison assay, 1 μg/ml of ^125^I-hTF was added to confluent HeLa cells, and incubated at 37°C for 0.5 and 3 hr. Cells were then washed with cold PBS, solubilized with 1 N NaOH, and radioactivity in cell lysate was determined. **B**. Uptake kinetics of rhTF and hTF in Caco-2 cells. One μg/ml of ^125^I-labeled hTF or rhTF was added to confluent Caco-2 cells and incubated at 37°C for 0.5, 1, 2, and 4 hr. Cells were then washed with cold PBS, solubilized with 1 N NaOH, and radioactivity in cell lysate was determined. The total cellular protein in the cell lysate was measured, and used to normalize the data to ng of hTF per mg cell protein (“ng/mg cell protein”). Data is represented as average with error bars indicating standard deviation, n = 3.

We have previously reported that the amount of endocytosed hTF increases slowly and is accumulated linearly in enterocyte-like Caco-2 cells, but reaches a plateau rapidly in most other cell lines including HeLa cells due to the rapid recycling and release [29]. To investigate if rhTF’s endocytosis is similar to that of hTF, the endocytosis kinetics of rhTF and hTF were assayed in HeLa and Caco-2 cells. It shows that the internalization of both hTF and rhTF increases rapidly and reaches a plateau after just 1 hr in HeLa cells (Figure 3A), while increases slowly and linearly up to the maximum 4 hr of our testing time in Caco-2 cells (Figure 3B). Furthermore, both the total uptake and the time-course kinetics of hTF and rhTF are similar regardless of the tested cell line (Figure 3). These results demonstrate that rhTF behaves similarly as hTF in the accumulation in Caco-2 cells and the rapid recycling pathway in HeLa cells.

#### Pulse-chase assay of rhTF in Caco-2 and HeLa cells

**Figure 4:**
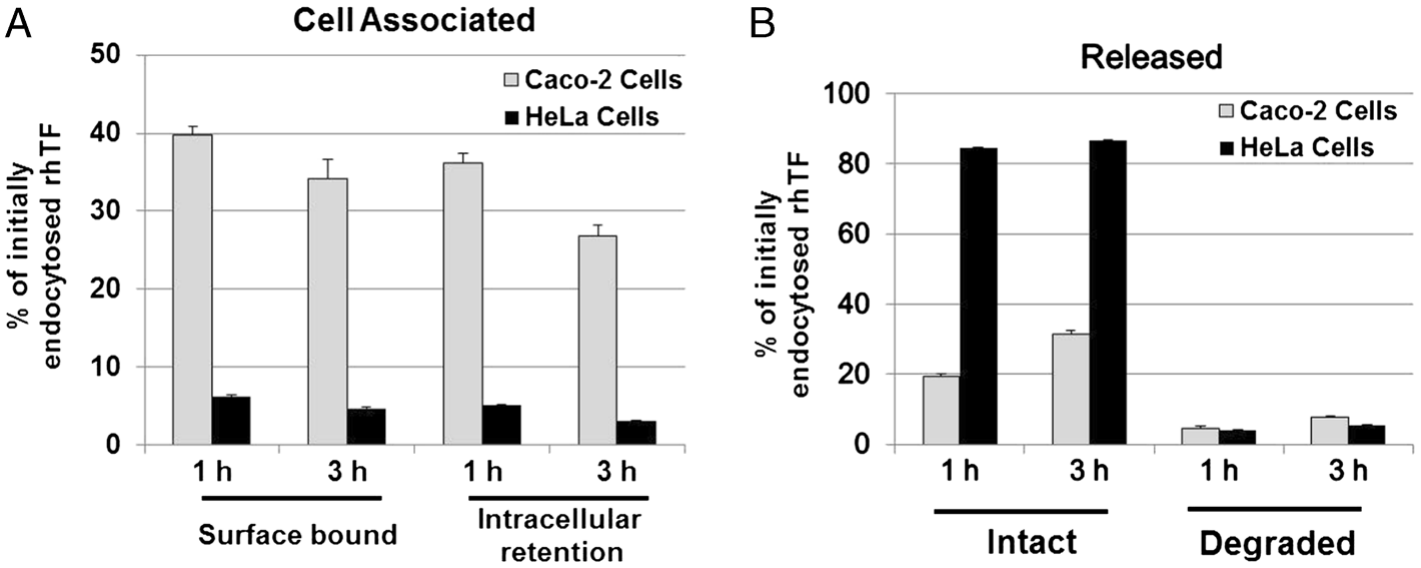
**Pulse Chase Assay of rhTF in Caco**-**2 and HeLa Cells.** Confluent Caco-2 or HeLa cells were pulsed for 1 hr by incubation with pulse medium containing 3 μg/ml ^125^I-labelled hTF or rhTF at 37°C. Then, the pulse medium with radiolabeled TF was removed by aspiration of the medium followed by washing cell monolayers with cold DMEM medium supplemented with 0.1% BSA. Caco-2 and HeLa cells were then chased by incubation with excess molar (0.3 mg/ml) of unlabeled hTF or rhTF in chase medium at 37°C. After chasing for 1 or 3 hr, the percentage of cell-associated (**A**) and released (**B**) hTF or rhTF was determined. Data is represented as average with error bars indicating standard deviation, n = 3.

We then carried out pulse-chase assays to further evaluate the endocytosis and intracellular processing of the rhTF protein in Caco-2 and HeLa cells. Following an 1 or 3 hr chase period, the majority of rhTF is still associated with the Caco-2 cells either intracellularly or surface bound (Figure 4A), whereas the majority of rhTF is released in HeLa cells and only about 11% of rhTF remain cell associated (Figure 4B). This result is consistent with the above finding via the rhTF endocytosis kinetics that rhTF is recycled rapidly in HeLa cells but accumulated intracellularly in Caco-2 cells.

**Figure 5:**
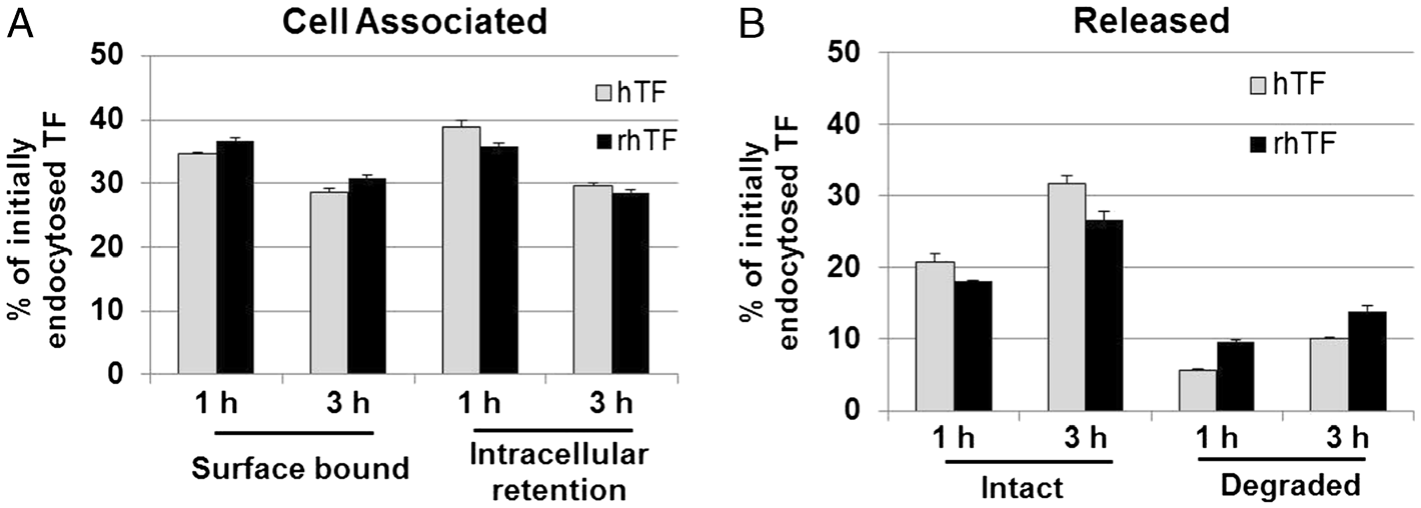
**Pulse Chase Assay comparing hTF and rhTF in Caco**-**2 cells.** Confluent Caco-2 cells were pulsed for 1 hr by incubation with pulse medium containing 3 μg/ml ^125^I-labelled hTF or rhTF at 37°C. Then, the pulse medium with radiolabeled TF was removed by aspiration of medium followed by washing cell monolayers with cold DMEM medium supplemented with 0.1% BSA. Caco-2 cells were then chased by incubation with excess molar (300 μg/ml) of unlabeled hTF or rhTF in chase medium at 37°C. After chasing for 1 or 3 hr, the percentage of cell-associated (**A**) and released (**B**) hTF or rhTF were determined. Data is represented as average with error bars indicating standard deviation, n = 3.

To have a direct comparison of rhTF and hTF for their endocytosis and intracellular processing, pulse-chase assays of rhTF and hTF were performed in Caco-2 cells. After Caco-2 cells were pulsed with the ^125^I-labeled rhTF or hTF for 1 hr followed by being chased with excess amount of unlabeled rhTF or hTF for 1 or 3 hr, the amount of cell-associated and released ^125^I-rhTF or hTF were determined to track the movements of TF in Caco-2 cells (Figure 5). After a 1 hr chase, the amount of ^125^I-rhTF or hTF protein remaining inside the cells is about the same as the amount associated with the cell surface (~35%) (Figure 5A). Of the ~30% of the ^125^I-rhTF or hTF that was released into the chase media, the majority was released intact (Figure 5B). After a longer chase period of 3 hr, both the intracellular and surface bound ^125^I-rhTF and hTF decreased to ~30% (Figure 5A), and the decrease corresponded with a proportional increase in the amount released into the chase media (Figure 5B). It shows that both the recycling and intracellular accumulation of rhTF in Caco-2 cells is similar to that of hTF. These results, taken all together, indicate that the retention and recycling of rhTF in Caco-2 and HeLa cells is similar to that of hTF.

### Cellular iron delivery ability of rhTF to support cell proliferation and antibody production in mammalian cells

Iron is absolutely required to sustain mammalian cell growth and proliferation and is essential for such processes as electron transfer, oxygen transport, and DNA synthesis [30,31]. We used two diverse cell lines (HL-60 and Sp2/0 hybridoma) to determine whether rhTF has similar biological activity as serum hTF to support cell growth and function. Furthermore, we measured the production of antibody from hybridoma cells as an indication of normal cellular function.

**Figure 6:**
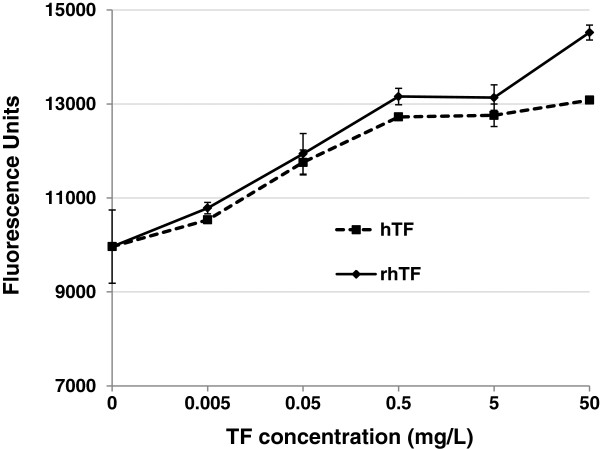
**Effect of rhTF on the proliferation of HL**-**60 cells.** The viable cell concentration of HL-60 cells after three days culture in serum-free medium supplemented with no hTF, 0.005, 0.05, 0.5, 5 and 50 mg/L of hTF (holo-form, from Sigma) or rhTF (Partial; partially iron saturated) was determined by fluorescence assay. Data is represented as average with error bars indicating standard deviation, n = 3.

HL-60 cells have been reported to have an absolute requirement of transferrin and insulin for cell proliferation [32], and have been widely used as a model system for the study of myeloid differentiation, hematopoiesis, and acute promyelocytic leukemia [33-35]. A dose response comparison was performed with varying concentrations of both hTF and rhTF to support the growth of HL-60 cells in a serum-free culture system. As shown in Figure 6, partially iron saturated rhTF and human holo-hTF produced a similar degree of cell proliferation at each comparative concentration. These data indicate that rhTF has similar potency to hTF and can deliver necessary iron to HL-60 cells similarly as hTF.

**Figure 7:**
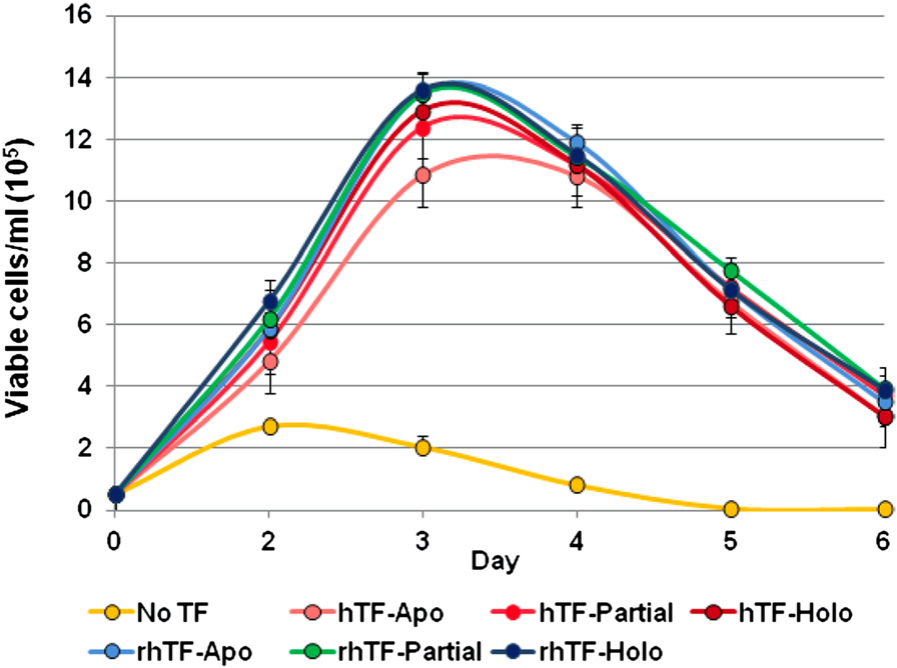
**The growth kinetics of hybridoma cells in serum**-**free medium supplemented with hTF or rhTF at 5 mg/L.** The hTF and rhTF were compared at different degrees of iron saturation at 5 mg/L concentration. Apo, iron-free; Partial, partially iron saturated; Holo, iron saturated. Error bars denote the standard deviation of triplicate cultures.

To investigate rhTF’s effectiveness of delivering iron to Sp2/0 hybridoma cells to stimulate cell proliferation and antibody production, we assayed three forms of rhTF or hTF, each at different degrees of iron saturation, i.e., iron-free (apo), partially iron saturated, iron-saturated (holo). We first compared the growth kinetics of hybridoma cells in serum-free medium supplemented with the 3 forms of hTF or rhTF at 5 mg/L over 6 days of culture (Figure 7). It shows that cells proliferated at a greatly reduced rate through the initial 2 days and failed to proliferate beyond two days of culture in medium without TF (Figure 7, Yellow), suggesting that the little amount of serum-derived transferrin in the cells is not sufficient to support a robust growth. However, robust growth of cells was enabled upon the addition of transferrin to serum-free medium. Hybridoma cells grew in log-phase through day 3, reached stationary phase on day 3–4, and entered death phase on day 5–6 (Figure 7). Furthermore, cells grown in medium supplemented with each of the 3 forms of rhTF showed similar growth kinetics as cells grown in medium supplemented with the 3 forms of hTF. Thus, rhTF and hTF resulted in similar, robust, growth kinetics regardless of their respective degree of iron saturation.

We then performed a dose response study to compare the activity of the hTF and rhTF using three cell culture performance metrics: 1.) log –phase cell proliferation after 3 days of culture, 2.) sustained cell growth as measured by cumulative cell density through 6 days of culture, and 3.) antibody productivity at the end-of-batch culture on day 6.

**Figure 8:**
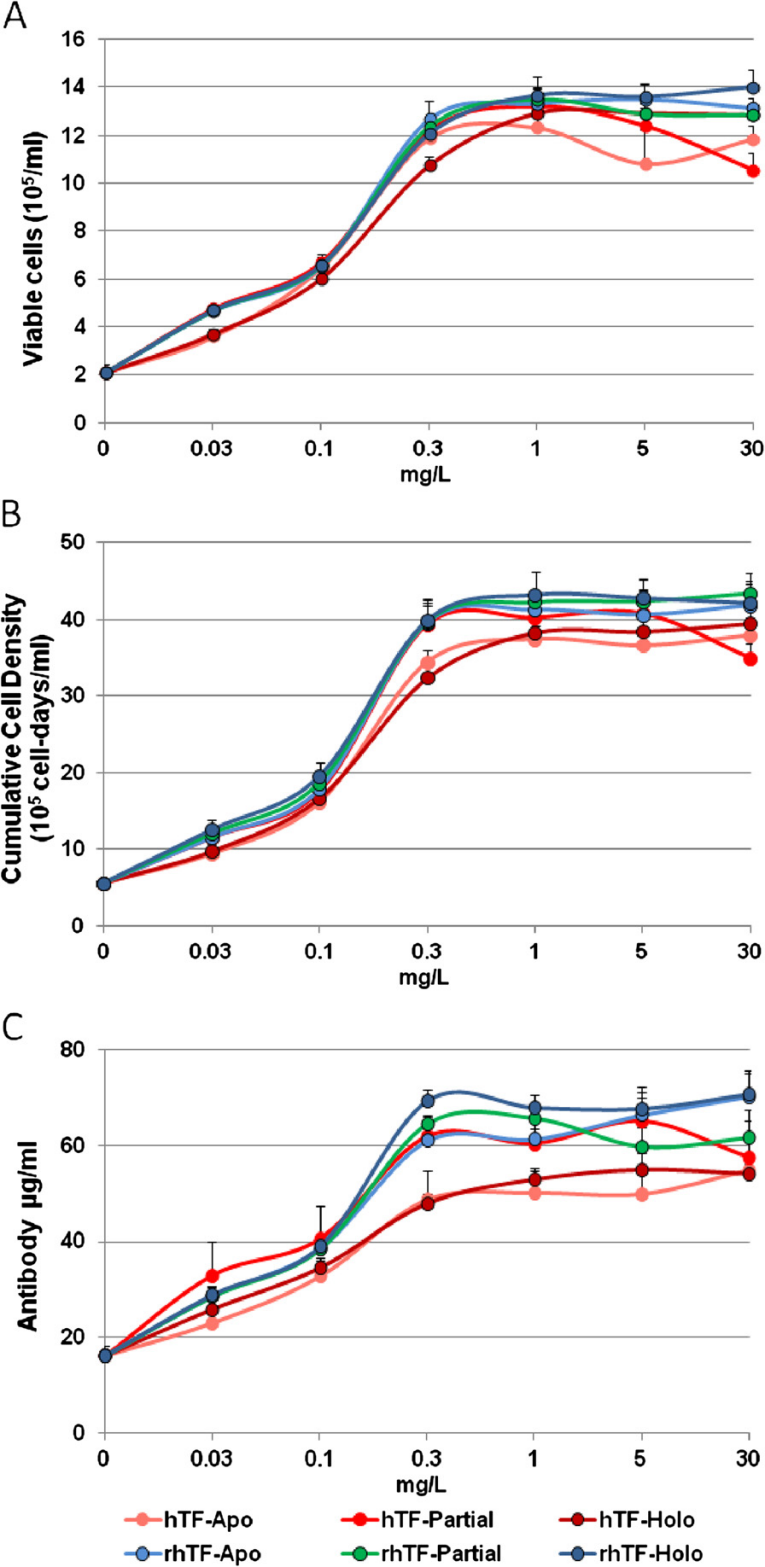
**Comparison of rhTF and hTF for supporting hybridoma cell proliferation**, **cumulative cell density, and antibody production.** Sp2/0 hybridoma cells were cultured in serum-free medium supplemented with hTF or rhTF at 0.03, 0.1, 0.3, 1, 5, or 30 mg/L. The hTF and rhTF were compared at different degrees of iron saturation: Apo, iron-free; Partial, partially iron saturated; Holo, iron saturated. **A**. Comparison of hTF to rhTF for cell proliferation during log phase growth (day0 through day3). **B**. Comparison of hTF to rhTF for cumulative cell density. Cumulative cell density is the estimated area under the growth curve and is indicative of the total cell mass generated by a culture system. Units are given in cell-days/ml. **C**. Comparison of hTF to rhTF for antibody production. Error bars denote the standard deviation of triplicate cultures.

Figure 8A shows the dose response comparison of the transferrins to support log-phase cell proliferation. We found that rhTF and hTF exhibit the same dose response curve to support cell proliferation regardless of their respective degree of iron saturation.

Furthermore, we compared rhTF to hTF using the parameter of 6-day cumulative cell density (CCD), also known as IVC-integral of the viable cell concentration. CCD is an important cell culture performance metric that measures the ability of a cell culture medium to support sustained high density cell growth [36] throughout an entire batch process. As shown in Figure 8B, rhTF and hTF have the similar CCD values at their equivalent concentrations. However, the generation of CCD was independent of the iron saturation status of both rhTF and hTF.

The concentration of monoclonal antibody produced by Sp2/0 cells grown in medium supplemented with a range of concentrations of rhTF or hTF at different degrees of iron saturation was compared (Figure 8C). rhTF and hTF stimulated the production of a similar amount of antibody at their equivalent concentration, and the production of antibody increased to the same extent with the increment dose for all of the transferrins tested. These results suggest that rhTF is equivalent to hTF for the delivery iron to hybridoma cells as evidenced by enhanced cell proliferation and the support of the cell’s physiological function of antibody production.

## Discussion

The essential function of TF is to transport and deliver irons to cells through the unique TF-TFR complex-mediated endocytosis pathway [1,3]. Our previous cell-free biochemical and biophysical studies show that both lobes of rhTF bind Fe^3+^ tightly yet reversibly similarly to hTF [15,25]. In this study we further characterize the TFR binding, TFR-mediated endocytosis and iron delivery function of rhTF using cell-based assays.

Our data show that the TFR-mediated endocytosis and intracellular processing of rhTF, as evaluated by total cell uptake, kinetics, and pulse-chase assays, is similar to that of hTF. This result is consistent with our previous finding in a competitive immunoassay that rhTF is equivalent to baby hamster kidney (BHK) cell derived recombinant N-His hTF in its ability to bind to the soluble portion of the TFR [15]. It is also in good agreement with other reports that hTF’s internalization increases linearly with time in enterocyte-like Caco-2 cells due to this type of cells’ unique accumulation of TF [29]. These results indicate that rhTF is similar to hTF in its ability of binding to human TFR, and then being endocytosed through TFR-mediated pathway. While the *in vitro* cell-based assays demonstrate the similarity of cellular iron delivery processing of rhTF and hTF, we have carried out a preliminary *in vivo* study to compare the serum half-life of rice-derived rhTF with that of hTF and the yeast-derived aglycosylated rhTF (CellPrime^TM^ rTransferrin AF, Millipore). The elimination half-lives (i.e. β-phase) of rice-derived rhTF, yeast-derived aglycosylated rhTF and hTF are 14.8, 13.8, and 18.6 hr, respectively (Unpublished data). The relatively shorter serum half-life could be due to the lack of N-linked glycans in the two recombinant transferrins [14,15,25]. Nevertheless, rice-derived rhTF is shown to have a sufficiently long serum half-life compared to native hTF. These results support that rhTF can be used as an animal-free alternative to serum hTF for pharmaceutical applications as a carrier to a number of drug molecules for drug targeting and delivery [6-8,37].

We also use cell culture assay to assess the functional equivalency of rhTF to hTF. We demonstrate that rice-derived rhTF is equivalent to hTF to carry out cellular iron delivery for supporting cell growth and differentiation of HL-60 cells, which have been described as dependent on TF to support cell proliferation [32]. Similarly, we show that rhTF and hTF has the same ability to deliver iron to support the cell growth and antibody production of Sp2/0 hybridoma cells, which are widely used for production of therapeutic antibodies [38]. In addition, a study by D-Finitive Cell Technologies also indicates rhTF has equal activity to hTF and iron chelate to improve the expansion of both mononuclear cells and CD34+ stem cells (Paul Price, unpublished data). All these results demonstrate that rhTF is same as hTF to be able to deliver irons to various mammalian cells for supporting cell physiological function.

Our data show that the iron saturation status of rhTF or hTF has little impact on the stimulation of cell proliferation and antibody production in the HL-60 and murine hybridoma cells. Although apo-TF has no bound iron to deliver to cells and has low affinity for TFR [3], its equivalent stimulation effect on cell proliferation and antibody production as holo-TF is most likely due to the availability of iron in culture medium and the binding of iron by apo-TF. Iron is an ingredient in many classical formulations including MEM, DMEM, alpha MEM, M199, and DMEM/F12, and thus apo-TF is likely becoming saturated with iron from the medium.

## Conclusion

Rice-derived rhTF is shown to be similar to hTF in its TFR binding, TFR-mediated endocytosis and cellular iron delivery function. This functional similarity, together with our previous reports showing the structural and biochemical similarities, makes rice-derived rhTF a low-cost and animal-free alternative to plasma-derived hTF for bioprocessing and biopharmaceutical applications. Currently, cell culture grade rhTF (Optiferrin^TM^) is available for use in cell culture applications. A more highly purified biopharmaceutical grade of rhTF is also being developed at Ventria Bioscience for use in pharmaceutical applications such as being used as a potential conjugate carrier to a number of drug molecules, including various chemotherapy drugs for drug targeting and delivery.

## Methods

### Materials

Recombinant hTF was expressed and purified from transgenic rice grains as described previously [25]. The native hTF, ferric ammonium citrate, bovine serum albumin (BSA), 4′-hydroxyazobenzene-2-carboxylic acid (HABA)/Avidin reagent, and horseradish peroxidase (HRP)-conjugated anti-goat IgG antibody, sodium selenite, and ethanolamine were obtained from Sigma (St. Louis, MO). Cell culture Dulbecco’s modified Eagle’s medium (DMEM) and Fetal bovine serum (FBS) were products of Mediatech (Manassas, VA). Dulbecco’s modified Eagle’s medium-Ham F-12 nutrient mixture (DMEM/F12) was purchased from Life Technologies. Trypsin-EDTA was purchased from Gibco BRL (Rockville, MD). The Na-^125^I was from Perkin Elmer (Waltham, MA). The BCA (bicinchoninic acid) protein assay kit, Sulfo-NHS-LC-Biotin, and PBS reagents (NaCl, KCl, Na2HPO4, KH2PO4) was from Thermo-Fisher (Waltham, MA). The culture dishes were products of Corning (Corning, NY). Avidin-coated plates were products of Roche. TMB (3,3′,5,5′-Tetramethylbenzidine) Microwell Peroxidase Substrate System was the product of KPL (Gaithersburg, MD). Cellastim™ rhAlbumin is a product of InVitria (Fort Collins, CO). All other chemicals that are not specified above were purchased from Sigma.

HeLa human cervic carcinoma cells (CCL-2), Caco-2 human colon carcinoma cells (HTB-37), HL-60 human promyelocytic leukemia cells (CCL-240) and murinehybridoma cells, derived from a Sp2/0-Ag14 myeloma fusion partner (HB-72) were obtained from American Type Culture Collection (Rockville, MD, USA). All cell lines were maintained in DMEM/F12 medium supplemented with 10% FBS. Prior to assay, cells were washed three times with serum-free DMEM/F12 medium without TF.

### Preparation of radiolabeled hTF and rhTF

Prior to radiolabeling, both rhTF and hTF were first saturated with iron by incubating 10 mg protein with 10 mg ferric ammonium citrate at 37°C in 2 mL of PBS, pH 7.2, for 2 hr followed by dialyzing against 2 L of PBS overnight at 4°C. Then, the iron-saturated TF was iodinated using the chloramines-T method [39]. The specific activities of ^125^I-TF ranged from 400 to 900 cpm/ng.

### TFR-binding affinity of rhTF

The TFR-binding affinity of rhTF was assessed using Caco-2 and HeLa cells. The Caco-2 and HeLa cells were adapted to growth as reported by Grasset et al. [40] and Zaro et al. [41], respectively, and then were seeded in a 12-well cluster plate to obtain confluent cell monolayers within a week after passage. Prior to TFR binding assay, Caco-2 and HeLa cells were washed twice with serum-free medium at room temperature and then pre-incubated with serum-free medium with 1 mg/ ml BSA at 37°C for 1 hr to deplete endogenous TF.

#### TFR-competition binding affinity assay in Caco-2 cells

One μg/ml ^125^I-hTF was mixed with 0.1, 0.3, 1, 3, 10 or 30 μg/ml unlabeled hTF or rhTF, respectively, and added to confluent Caco-2 cells followed by incubation at 4°C for 2 hr. The medium was then aspirated, and the cells were washed with ice-cold PBS and then solublized in 1 M NaOH. The amount of ^125^I-hTF and the cell protein content in the lysates were measured using a gamma counter and the BCA assay kit (Pierce), respectively. The non-specific surface binding of ^125^I-hTF was determined in wells containing cells supplemented with a 100-fold excess of unlabeled hTF or rhTF. The amount of ^125^I-hTF bound to TFR was calculated by subtracting non-specific surface bound ^125^I-hTF from the total amount of ^125^I-hTF in the lysates.

#### TFR-binding affinity assay in HeLa cells

Saturation radio-ligand binding assays were performed in HeLa cells to determine the binding capacity (B_max_) and equilibrium dissociation constant (K_d_) values of rhTF. Increasing concentrations of ^125^I-hTF or rhTF (0.1, 0.3, 1, 3, or 10 μg/ml) were added to the confluent HeLa cells followed by incubation at 4°C for 2 hr. In parallel, cells incubated with a 100-fold excess of unlabeled hTF or rhTF was used to normalize the background caused by non-specific binding. Then, the TFR-binding affinity was assessed by the same method as described above in section TFR-competition binding affinity assay in Caco-2 cells.

### TFR-mediated endocytosis of rhTF

The TFR-mediated endocytosis of rhTF was assessed using Caco-2 and HeLa cells, and the culture of these two cells was the same as described above in TFR binding affinity assay.

#### Comparison of endocytosis of rhTF in HeLa and Caco-2 cells

One μg/ml ^125^I-rhTF was added to confluent HeLa or Caco-2 cells followed by incubation at 37°C for 0.5, 1, 2, or 4 hr. In parallel, a 100-fold excess of unlabeled hTF was added to the wells containing ^125^I-rhTF to assay the non-specific binding of the ^125^I-rhTF. The medium was aspirated, and the cells were washed with ice-cold PBS and solublized in 1 M NaOH. The radioactivity of the lysates was counted using a gamma counter, and the cell protein content was determined using the BCA assay to determine the total cellular uptake of ^125^I-rhTF. TFR-mediated cellular uptake of rhTF was calculated by subtracting non-specific surface binding of rhTF from the total cellular uptake of rhTF.

#### Comparison of endocytosis of rhTF and hTF in Caco-2 cells

To investigate if the cellular uptake of rhTF is same as its native counterpart hTF, 1 μg/ml ^125^I-rhTF or hTF was added to confluent Caco-2 cells followed by incubation at 37°C for 0.5, 1, 2, or 4 hr. Then, the internalized ^125^I-rhTF or hTF in Caco-2 cells was assayed with the same method as described above for comparison of endocytosis of rhTF in HeLa and Caco-2 cells.

#### Pulse-chase assays of rhTF in HeLa and Caco-2 cells

Confluent HeLa and Caco-2 cells were first incubated with pulse medium containing 3 μg/ml ^125^I-rhTF at 37°C for 1 hr. In parallel, non-specific binding of ^125^I-rhTF was determined in wells containing ^125^I-rhTF and a 100-fold excess of unlabeled hTF. The unbound ^125^I-rhTF was then removed by three washes with serum-free medium. The pulse medium was aspirated, and the cell monolayers were washed with cold DMEM medium supplemented with 0.1% BSA to remove residual pulse medium. Chase medium, which contains 0.3 mg/ml unlabeled hTF to prevent re-internalization of ^125^I-rhTF, was then added to cells followed by incubation at 37°C for 1 or 3 hr. To determine the percentage of intact recycled ^125^I-rhTF in the chase medium, the collected medium was treated with 15% trichloroacetic acid for 15 min at 4°C. The medium samples were centrifuged, and the radioactivity in the pellet (intact) and supernatant (degraded) was determined. Meanwhile, the cell monolayers were incubated with trypsin, which detaches the cells and removes surface bound TF, and centrifuged to separate the surface bound (supernatant) and intracellular (pellet) ^125^I-rhTF. The data were presented as a percentage of initially endocytosed ligands (sum of release, surface-bound, and intracellular retention).

#### Pulse-chase assays of rhTF and hTF in Caco-2 cells

Confluent Caco-2 cells were first incubated with pulse medium containing 3 μg/ml ^125^I-hTF or rhTF at 37°C for 1 hr. Then, the endocytosis processing of radiolabeled rhTF and hTF in Caco-2 cells were assayed with the same method as described above in pulse-chase assays of rhTF in HeLa and Caco-2 cells.

### The cellular iron delivery ability of rhTF to support cell growth and antibody production in mammalian cells

Cell proliferation assay using human promyelocytic leukemia HL-60 cells was performed in DMEM/F12 medium supplemented with recombinant human insulin as described [32], and with either hTF or rhTF at 0.005, 0.05, 0.5, 5, or 50 mg/L. Cells were washed in DMEM/F12 medium and seeded at 5,000 viable cells per well in a 96 well plate with triplicate wells per condition. Following a 3 day incubation, the relative viable cell count was determined by Resazurin (alamarBlue®) fluorescence assay [42] and reported as fluorescence units (FU)

Cell growth and antibody production of Sp2/0 hybridoma cells were assessed in a serum-free medium composed of DMEM/F12 medium supplemented with 10 mg/L recombinant human insulin, 0.0067 mg/L sodium selenite, 2 mg/L ethanolamine, 1 g/L Cellastim™ rhAlbumin from InVitria (Fort Collins, CO). A series of increasing concentrations of rhTF or hTF at 0.1, 0.3, 1, 3, 10, or 30 mg/L were used for the comparison of rhTF to hTF. Washed hybridoma cells were seeded in a 6 well plate at 0.5 x 10^5^ viable cells/ml in triplicate 4 ml cultures. Cells were maintained in a humidified incubator at 37°C with 6% CO_2_. The concentration of viable cells was determined daily for 6 days by flow cytometer (Guava, Millipore) until end of batch culture was obtained (cell viability of 50% or less). The concentration of IgG1 monoclonal antibody secreted into the medium after 6 days of culture was determined by a fluorescence-based ELISA developed by InVitria using phycoerythrin conjugated detector antibody (Jackson ImmunoResearch, W. Grove, PA).

## Abbreviations

TF: Transferrin; hTF: Human serum transferrin; rhTF: Recombinant human serum transferrin; TFR: Transferrin receptor.

## Competing interests

The authors declare no competing interests.

## Authors’ contributions

DZ, NH and WCS designed the study, analyzed the data and wrote the paper. HFL and JLZ performed the TFR binding, TFR-mediated endocytosis experiments. SCP performed the cell culture experiments and analyzed the related data. All authors contributed to the manuscript and have read and approved the final manuscript.
